# Short-Term Efficacy of Pulsed Radiofrequency Thermal Stimulation on Acupoints for Chronic Low Back Pain: A Preliminary Study of a Randomized, Single-Blinded, Placebo-Controlled Trial

**DOI:** 10.1155/2018/4510909

**Published:** 2018-08-12

**Authors:** Boncho Ku, Minho Jun, Jun-Hwan Lee, Young-Ju Jeon, Young-Min Kim, Jaehui Kang, Yu-Jung Lee, Kahye Kim, Hyun Heo, Jaeuk U. Kim

**Affiliations:** ^1^Future Medicine Division, Korea Institute of Oriental Medicine, Daejeon 34054, Republic of Korea; ^2^Clinical Medicine Division, Korea Institute of Oriental Medicine, Daejeon 34054, Republic of Korea; ^3^Korean Medicine Life Science, University of Science & Technology (UST), Campus of Korea Institute of Oriental Medicine, Daejeon 34054, Republic of Korea; ^4^Spinal and Joint Center, Cheonan Oriental Hospital of Daejeon University, Cheonan 31099, Republic of Korea; ^5^Technology Licensing and Commercialization Team, Korea Institute of Oriental Medicine, Daejeon 34504, Republic of Korea; ^6^Solco Biomedical Co., Ltd., Pyeongtaek, Gyeonggi 17704, Republic of Korea; ^7^Department of Biomedical Engineering, Yonsei University, Wonju, Gangwon 26493, Republic of Korea

## Abstract

**Background:**

The objective of this study was to evaluate the pain-relief efficacy of thermal stimulation induced by a pulsed radiofrequency (PRF) thermal stimulation applied to acupoints (APs) in patients with low back pain (LBP). The study was designed as a randomized, single-blinded, placebo-controlled trial*. Methods.* Fifty-six LBP patients whose minimum pain intensity score on a visual analogue scale (VAS, 0-100 mm) was more than 30 mm were randomly allocated to either the placebo-controlled or the treatment group at a 1:1 ratio. The treatment and placebo-controlled groups received PRF thermal stimulation plus cupping therapy and cupping therapy only, respectively. Each patient was scheduled to receive a total of three treatment sessions over one week with allowing a window up to 4 days. Six of the 13 predefined APs were selected differently for each session depending on the change in patient's symptoms and intensity of pain. The primary outcome was the mean difference between the placebo-controlled and treatment group of VAS changes from the baseline to the end of the follow-up period.

**Results:**

The patients' reported VAS scores from baseline to the end of follow-up (average: 9.8 days) were significantly decreased by 8.036 points (two-sided 95% CI, -11.841 to -4.231) and 13.393 points (two-sided 95% CI: 17.198 to -9.588) in the treatment and the placebo-controlled groups, respectively. However, the change in VAS scores between the treatment group and the placebo-controlled group was not significantly different (2.015 mm, two-sided 95% CI: -5.288 to 9.317).

**Conclusion:**

The trial results indicated that treatment with either PRF thermal stimulation with cupping therapy or cupping therapy alone effectively relieved LBP. The efficacy of PRF thermal stimulation combined with cupping therapy was not superior to that of cupping therapy alone.** Trial registration number: **Clinical Research Information Service (KCT0002137). The trial was registered retrospectively on 10 November, 2016.

## 1. Introduction

Low back pain (LBP) has become a prevalent health problem in many economically developed countries [1]. More than 70% of the population in such countries has experienced LBP at some point in their lifetime, and the prevalence of chronic LBP is approximately 10 to 15% [2]. Due to the high prevalence of LBP, it is no longer considered a specific disorder limited to highly industrialized countries but is now considered a major health problem worldwide [2–4]. In particular, according to a survey conducted in 2007 among the adult population in South Korea, the number of patients who suffered from LBP was estimated to be greater than 5 million, and approximately 55% of these patients developed chronic LBP [5]. LBP causes large burdens in terms of medical expenses, work absences, and disability [6]. For example, Kim et al. [7] reported that LBP was the most common disease for workers' compensation losses accounted for up to 40 % of cost.

Although conventional approaches for the management of LBP such as spinal manipulation, analgesics, nonsteroidal anti-inflammatory drugs, muscle relaxants, and many other treatments are available, no single therapeutic approach appears to be superior to other modalities [8]. Because conventional therapeutic interventions are often ineffective [9, 10] and are accompanied by adverse effects that lead to the dissatisfaction of patients [11], the use of complementary and alternative medicine (CAM) to manage LBP has been highlighted and has increased over the last two decades [12, 13]. Various CAM modalities, such as acupuncture, massage, and exercise, have been applied to alleviate LBP, although the precise mechanisms of action of each treatment remain ambiguous and their efficacies in reducing pain and disability are inconsistent or are based on low-quality evidence [14–20]. Nevertheless, numerous researchers and practitioners have sought to demonstrate the efficacy and mechanism of action of such treatments based on the perspectives of modern science [21]. Among these efforts, several systematic reviews and meta-analyses of randomized control trials (RCTs) have revealed some evidence for the efficacy and safety of CAM therapies for LBP [12, 21–26].

The most commonly applied therapeutic method among the various CAM modalities for the management of LBP is acupuncture. The efficacy of acupuncture in mitigating LBP has been consistently reported, and its safety is generally accepted [11, 21, 25, 27, 28]. Other types of CAM therapy, such as moxibustion and cupping, are also used alone or in combination with acupuncture to alleviate musculoskeletal pain [29–31]. Moxibustion is a therapeutic method that involves applying heat stimulation to APs by burning herbal powder primarily consisting of mugwort (moxa,* Artemisia argyi*) [29]. Cupping therapy is an ancient TCM modality that generates negative pressure, inducing hyperaemia or homeostasis, at acupoints using cups composed of various materials, such as bamboo or glass [30, 32, 33]. Both therapies have been generally accepted to be effective in improving blood circulation and alleviating pain [34, 35]. Experimentally, heat and negative pressure on the surface of the skin have been reported to induce similar physiological responses; both modalities induce the dilation of local blood vessels, increase local circulation and microcirculation, promote angiogenesis, and remove chemical substances that sensitize nociceptors [30, 36, 37]. Although moxibustion and cupping therapy are widely applied to alleviate LBP, their safety have not been well investigated. Especially, moxibustion may induce unexpected adverse effects, including air pollution, epidermal burning, blistering, suppuration, infection, and bruising, mainly due to the difficultly in controlling the magnitude of heat intensity [38].

Radiofrequency (RF) current has been used as a treatment modality to manage chronic pain syndromes such as chronic cervical pain, brachialgia, and cervicogenic headache, and cancer pain [39, 40]. In comparison to the conventional continuous RF (CRF) [41, 42], pulsed RF (PRF) does not generate thermal damage of nervous tissues by allowing time for heat dispersion [43]. Recently, PRF is regarded as a safe and less-destructive modality for the management of pain such as shoulder pain, lumbar facet joint pain, and various type of neuropathic pain [43–46]. A mechanism of PRF in pain relief is still unclear. Up to date, most studies related to biological effects of PRF postulate that a type of neuromodulatory effect induced by alternating electrical field inhibits synaptic transmission and neuron-specific gene expression [42, 43, 45, 47, 48]. Even though PRF and moxibustion are not directly linked in terms of clinical mechanism, both therapeutic modalities generate local thermal stimulation penetrating the subcutaneous skin layer [49]. In this respect, the PRF-based thermal stimulation system is often used as an alternative to the traditional moxibustion [50, 51].

Thus far, the available commercial products related to CAM therapeutic devices are primarily acupuncture-like devices that replace acupuncture needles, such as low-intensity lasers, electrical stimulators, or focused magnetic field generators [52]. In contrast, devices designed for use in moxibustion or cupping have rarely been reported. Recently, Myoung et al. [51] developed a temperature-controllable PRF probe generating heat distribution similar with moxibustion. Based on their research, a device that simultaneously applies thermal stimulation based on PRF electric fields and cupping therapy was developed for clinical use. In this study, we conducted a conventional RCT including LBP patients to assess the short-term pain-relieving effect of a newly developed PRF thermal stimulator.

## 2. Methods

### 2.1. Study Design

A randomized, single-blinded, and placebo-controlled trial was performed in 2013 at the Spinal and Joint Center, Cheonan Oriental Hospital of Daejeon University, Republic of Korea. This study was conducted in parallel with another study evaluating the effectiveness of a laser acupuncture device, previously reported by Shin et al. [53]. The design of this study was similar to the design of the study by Shin et al. except that patients were independently recruited.

### 2.2. Ethics Approval

This study was approved by the Institutional Review Board of Cheonan Oriental Hospital of Daejeon University, Korea (M2013-03-2, registered on 1 November, 2013). This study was regulated by the Ministry of Food and Drug Safety (No. 416, registered on 1 October, 2013), and a trial registration number was retrospectively obtained from the Clinical Research Information Service (KCT0002137, registered on 10 November, 2016), retrospectively.

### 2.3. Participants

Patients aged from 20 to 75 years with unspecified, uncomplicated, or chronic LBP were recruited through advertisements and bulletin board postings at the hospital. LBP was diagnosed based on patient's history, symptom, previous radiographic records (e.g. X-ray, and CT), and independent physical examinations. The investigators or physicians provided full explanations of the purpose of study, interventions, and possible adverse events and complications, and written informed consent was obtained from all study candidates. LBP patients with a minimum pain intensity score greater than 30 mm on a visual analogue scale (VAS, 0 to 100 mm) were included. Enrolled patients were excluded if they met any of the following conditions: required the aid of medical devices or attached implantable equipment that could be affected by electromagnetic fields, such as pacemakers or hearing aids; had unendurable pain, bone fractures, severe disc herniation, or spinal tumours; were taking drugs such as corticosteroids, anticonvulsants, or anti-inflammatory agents; were pregnant; experienced any adverse effects due to the physical stimulation therapy; exhibited cognitive or mental dysfunction; or had participated in other clinical trials within the last month. Eligible patients were scheduled to visit three times over one week to receive treatment with allowing a window of 4 days, and follow-up investigations were performed within one week after the completion of treatment.

### 2.4. Randomization

Eligible patients were randomly allocated to the PRF thermal stimulation plus cupping therapy treatment group or the cupping therapy alone placebo-controlled group at a 1:1 ratio. Balanced block randomization was performed using the blockrand() function in the* blockrand* package [54] that is provided in the current version of the R statistical package (R Core Team, Austria). The block size was randomly selected with lengths of 2, 4, and 6 for group allocation.

### 2.5. Blinding

Access to the results of the randomization table was strictly prohibited with the exception of the independent statistician. The group assignment result was delivered to the hospital in the form of an opaque envelope labelled with consecutive numbers. All relevant investigators, including clinical coordinators and practitioners, were blinded to the type of treatment until the end of the study. The allocation was conducted by opening the envelope sequentially in front of the patient immediately before the first intervention. The patients received only partial information that corresponded to the masked group assignment (labelled as group A or B), and their actual treatment was concealed during the study. The devices used in both the treatment group and the placebo-controlled group were manufactured with identical appearances, but the PRF irradiation output of the device used for the placebo-controlled group was not operational. The practitioners were only able to identify each device according to the masked group labels. Acoustic sounds mimicking the application of PRF thermal stimulation and the pressure of cupping on peripheral APs were also delivered by the devices to preserve the group blinding of the practitioners.

### 2.6. Interventions

**Figure 1:**
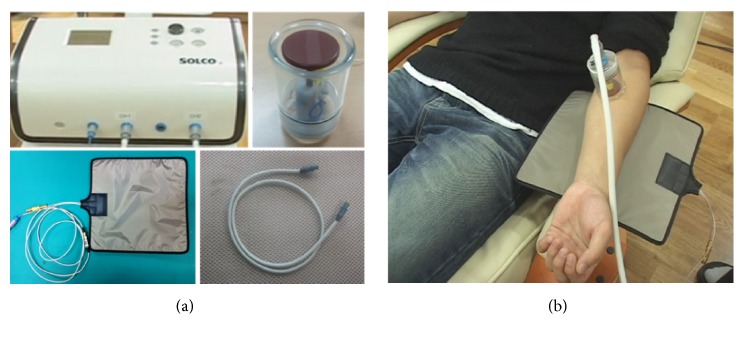
(a) Appearance and accessories of the PRF thermal stimulation device. From the left to right in a clock-wise direction: main body cup-shaped probe equipped with PRF radiation tip; connection cable; and electrode pad. (b) Example of the operation of the PRF thermal stimulation device.

Patients received a combination of PRF thermal stimulation and cupping therapy or only cupping therapy three times over the course of one week. The PRF thermal stimulation and cupping device (Solco-HF100, Solco Biomedical, Co., Ltd., Republic of Korea) consisted of the following components: a main body to control the magnitude of PRF stimulation, cup-shaped probes equipped with a PRF stimulator at the tip, a connection cable for the probe, and an electrode pad for PRF induction. The detailed appearance of each component of the device is shown in Figure 1(a). PRF of 2 MHz was applied at intervals of 2000 ms through channel 1, and the actual stimulation duration of each application was configured to 400 ms. The output power was initially set at 20 W (10% of the maximum output power), and the negative pressure was 15 kPa.

**Figure 2:**
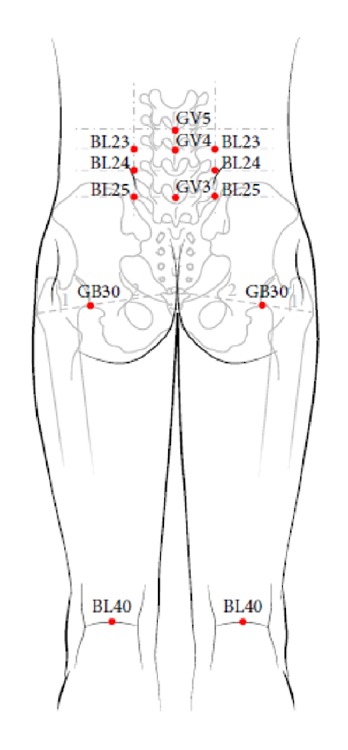
The location of the acupoints selected in the study. The figure was originated from the article of Shin and Jae-Young et al. “Short-Term Effect of Laser Acupuncture on Lower Back Pain: A Randomized, Placebo-Controlled, Double-Blind Trial,”* Evidence-Based Complementary and Alternative Medicine* 2015 [53].

In this study, an individualized acupuncture treatment for each LBP patient allowed flexible six APs within predefined APs depending on the patient's symptom progress and intensity of pain. Two licensed Korean Medicine doctors whose clinical experience was more than two years screened a total of 13 predefined APs based on their careful consensus and the literature [55]. The predefined APs include five bilateral (BL23, BL24, BL25, GB30, and BL40) and three unilateral (GV3, GV4, and GV5) points (see Figure 2). APs including BL23, BL24, BL25, GV3, GV4, and GV5 were selected because these APs were located at low back and LBP patients usually feel pain at those points. The rest of predefined APs, GB30 and BL40, was one of frequently selected APs to LBP treatment not only in South Korea also worldwide [56, 57]. In addition, all these APs are located where the contact is possible, reflecting the structural feature of the probe mounted on the PRF thermal stimulation system.

For the treatment group, PRF thermal stimulation and cupping therapy were applied to each of the patient-specific APs for ten minutes. In the placebo-controlled group, an identical treatment procedure was performed except that PRF thermal stimulation was not actually applied. An example of the use of the probes is depicted in Figure 1(b).

### 2.7. Concomitant Medications

Patients in both the treatment group and the placebo-controlled group were suggested to voluntarily and independently perform daily exercises for LBP. Other treatments or therapies related to ameliorating LBP were prohibited during the study with the exception of the guided therapies. All significant medications and nonmedication treatments that were administered to patients after enrolment were reported in the “concomitant medications/significant non-drug therapies after the start of study” form.

### 2.8. Outcome Measures

#### 2.8.1. Primary Outcome

The primary outcome measure was the change in LBP intensity measured before application of the intervention and one week after the end of the intervention. Patients were asked to score their subjective LBP intensity according to the 100 mm VAS. A decrease of VAS indicates the improvement of pain relief. The VAS score was evaluated on the first day of screening (visit 1), after each of the three interventions on visits 1 to 3, and at the follow-up visit. The difference of mean changes between placebo-controlled and treatment group is the primary concern in this study.

#### 2.8.2. Secondary Outcomes

The secondary outcomes included the pressure pain threshold (PPT), the patient global impression of change (PGIC), and the European Quality of Life-5 Dimensions (EQ-5D).

The PPT represents a cut-off point at which a nonpainful pressure stimulation changes to a painful pressure [58]. A digital pressure algometer (AA129, JTECH Medical, USA) was used to quantify the PPT. The investigator placed an algometer on both sides of each patient's BL25 point and increased the pressure (kg/cm^2^) applied perpendicular to the patient's skin. The pressure was increased until the patient noticed the first sensation of pain: each patient was instructed to raise a hand or announce when the pain was noticed. An increase of PPT indicates that the subject is more endurable to the pressure pain, so it can be interpreted that pain is alleviated. The PPT was evaluated on visits 1 to 3 and at the follow-up visit.

The PGIC is recommended tool to evaluate chronic pain in clinical trials [59]. The PGIC was adopted to assess the general improvement in the LBP of each patient after the intervention compared to before the intervention. The impression of this change was graded on a seven-point Likert scale (1: very much improved; 2: much improved; 3: moderately improved; 4: not improved; 5: slightly worse; 6: severely worse; or 7: very severely worse). The PGIC was reported on visit 1 and at the follow-up visit.

The EQ-5D is a representative instrument for measuring the patient's general health status and was developed by the EuroQol group [60]. The Korean translated version of the EQ-5D is composed of 5 dimensions: mobility, self-care, usual activities, pain/discomfort, and anxiety. Each dimension is graded on three levels and is scored using weights as estimated in a recent study of the EQ-5D for the Korean population [61]. The EQ-5D was assessed at visit 1 and at the follow-up visit.

### 2.9. Sample Size Calculation

Due to the limited existing evidence available to estimate the effect size of PRF thermal stimulation on LBP at the time of the study design, the sample size was determined based on previous results of a meta-analysis on the efficacy of acupuncture for LBP [27]. The pooled estimate of the effect size for the short-term effectiveness of acupuncture was 0.58 (95% CI: 0.36 – 0.80) based on four studies. It was necessary to conduct the preliminary study at minimal expense and, therefore, we intentionally overestimated the effect size and ultimately selected the value 0.80, which represented the upper bound of the 95% CI for the mean effect size. Using the above result, the sample size required to detect a significant difference in the primary outcome between groups on an independent two-sample t-test was 25 for each treatment group considering 80% power and a significance level of 5% (two-tailed). For a one-to-one group allocation ratio and allowing for a drop rate of 10%, a total of 56 patients were recruited.

### 2.10. Statistical Analysis

Statistical analyses were performed using R statistical software, version 3.1.0. The level of significance was set to 0.05, and two-tailed comparisons were performed. The analyses of all measures, including the primary and secondary outcomes, were conducted on the full analysis set (FAS) based on the principle of intention-to-treat. Per protocol subsamples were also analysed for comparison with the results derived from the FAS analysis. There were no significantly discordant results between the two analyses and, therefore, the results of the PP analysis are not shown. Missing data were imputed using the last observation carried forward method. The differences in baseline characteristics between the active and placebo PRF stimulation groups were evaluated using independent two-sample t-tests or Wilcoxon's rank sum tests for continuous variables and Chi-squared tests or Fisher's exact tests for categorical variables.

An analysis of covariance (ANCOVA) was employed to evaluate the differences in the primary and secondary outcome measures between the treatment and placebo-controlled groups. The change in VAS scores between baseline and the endpoint was analysed using ANCOVA with adjustments for the baseline score and predetermined confounding factors of sex, age, the duration of back pain, and the number of exercise therapies performed during the study. A linear mixed effects model using the lmer() function in the lme4 package (R statistical package) was applied to investigate changes within each treatment group for each visit compared to baseline for the primary and secondary outcomes.

## 3. Results

**Figure 3:**
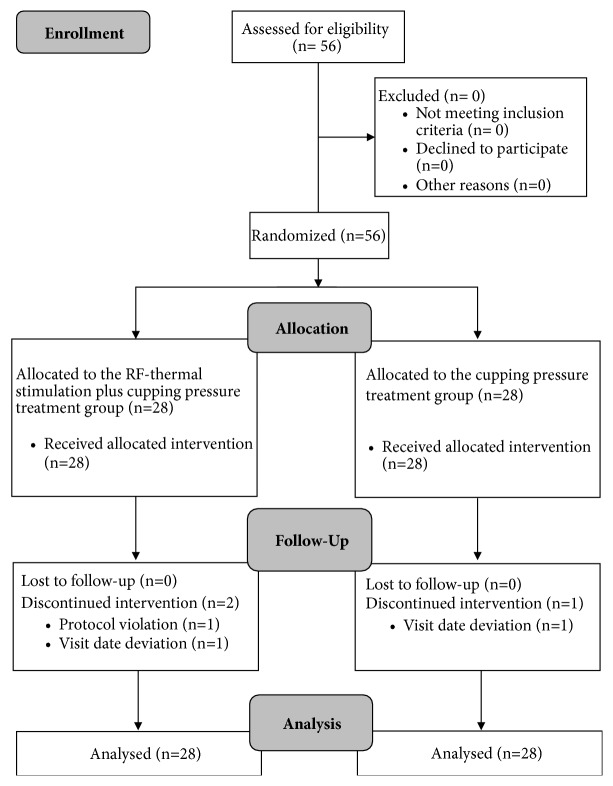
CONSORT flow diagram of the trial.

A total of 56 patients recruited during November to December 2013 were assessed for eligibility and were equally randomized to the active (n=28) and control (n=28) PRF stimulation groups. Two patients in the treatment group and one patient in the placebo-controlled group dropped out. One patient in the treatment group violated the protocol, and two patients (one from the active group and one from the control group) did not appear on the scheduled visit dates. A flow diagram describing the study is presented in Figure 3.

### 3.1. Baseline Characteristics

**Figure 4:**
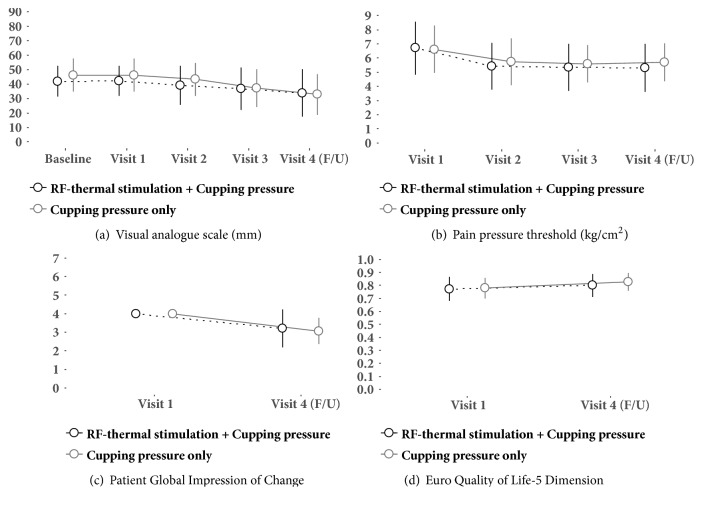
Crude mean profile of the primary and secondary outcomes (mean for each symbol and standard deviation for the error bars). Each panel represents (a) visual analogue scale (VAS), (b) pain pressure threshold (PPT), (c) patient global impression of change (PGIC), and (d) Euro Quality of Life-5 Dimension (EQol-5D).

The patients' demographics and baseline values for the main outcomes are summarized in Table 1. The samples of the two groups had equally balanced distributions: there were no significant differences between the treatment and placebo-controlled groups regarding sex ratio, age, duration of LBP, compliance with exercise guidance, body mass index, systolic/diastolic blood pressure, pulse and body temperature, or measured outcomes, including the VAS scores, the PPTs, and the EQol-5D results. The profile of the crude means and standard deviations of both primary and secondary outcomes measured in each session was illustrated in Figure 4.

### 3.2. VAS Change in LBP Intensity

The mean VAS scores were significantly reduced for both types of intervention (Table 2). After completion of the three intervention sessions over the course of one week, the changes in VAS scores at the time of follow-up were -8.036 (95% CI, -11.841 to -4.231; p<0.0001) for the PRF thermal stimulation plus cupping therapy group and -13.393 (95% CI, -17.198 to -9.588; p<0.0001) for the cupping therapy alone group. However, based on the between-group analysis, the adjusted mean difference in the change in VAS scores between the two groups at the time of follow-up was 2.015 (95% CI, -5.288 to 9.317; p=0.7090), and this difference remained insignificant throughout all intervention stages (Table 2).

### 3.3. Pressure Pain Threshold

Based on the within-group analysis, the mean changes in PPT at the time of follow-up were -1.421 (95% CI, -1.932 to -0.911; p<0.0001) and -0.935 (95% CI, -1.445 to -0.425; p=0.0012) in the treatment and placebo-controlled groups, respectively. Similar to the VAS score results, the adjusted mean differences in the change in PPT between the treatment and placebo-controlled groups were not significant during the intervention or at the follow-up visit (Table 2).

### 3.4. Patient Global Impression of Change

The mean change in the PGIC was significant at the time of follow-up in both groups: -0.786 (95% CI, -1.115 to -0.457; p<0.0001) in the treatment group and -0.929 (95% CI, -1.258 to -0.600; p<0.0001) in the placebo-controlled group. There was no significant difference in the adjusted mean difference in the change in PGIC between the two groups.

### 3.5. EQ-5D

The mean change in the EQ-5D result was 0.026 (95% CI, 0.000 to 0.052; p=0.0485) in the treatment group and 0.050 (95% CI, 0.024 to 0.076; p<0.0001) in the placebo-controlled group. There was no significant adjusted mean difference in the change in the EQol-5D result between the treatment and placebo-controlled groups (Table 2).

### 3.6. Safety Analysis

Although one patient in the placebo-controlled group complained of a mild sore throat during the study, the event was deemed irrelevant to the study. No adverse events directly related to the interventions were reported during the study.

## 4. Discussion and Conclusion

In the present randomized, double-blinded, placebo-controlled trial, we investigated the efficacy of PRF thermal stimulation using a newly developed device for modulating the functions of moxibustion and cupping therapy in patients with LBP. Combined treatment of PRF thermal stimulation and cupping therapy was not significantly different from treatment with cupping therapy alone based on the primary and secondary outcomes. Nevertheless, the results showed that the PRF thermal stimulation and cupping therapy induced by the developed device were safe treatments for LBP patients and provided a clinical advantage in alleviating LBP. In both the treatment and control groups, LBP was significantly alleviated at the time of the follow-up visit after the end of all planned interventions. The VAS score revealed that the patients experienced a significant reduction in pain, and other secondary outcomes, including PGIC and EQol-5D, also showed improvements. In the case of PPT, unlike the results of other outcomes, LBP was increased in both placebo-controlled and treatment groups. This result is also opposite to the previous studies [62, 63] reporting PPT as an outcome. To clarify this discrepancy, a well-designed study that minimizes experimental biases is required.

The present study design was similar to that of the study conducted by Shin et al. [53], except for the different sources of stimulation and of enrolled LBP patients. Recent systematic reviews of LBP that have investigated the efficacy of CAM therapies generally defined the duration of ‘short-term' follow-up as corresponding to an endpoint within two weeks to three months after treatment [11, 12, 27, 57]. The most common duration that patients used CAM therapies for treatment of a single disease in South Korea was reported to be approximately three to six days based on a survey conducted in 2009 [64]. In this respect, one-week duration selected for LBP treatment was of practical reason. The APs of BL23, BL25, BL40, GV3, GV4, and GB30 are globally used for the management of nonspecific and chronic LBP according to both textbook and clinical practice, as reviewed by Yuan et al. [57]. In addition, in a recent study, the result of a network analysis indicated that the set of APs applied in this study represented the most typically used points for treating LBP and that those 13 APs tended to be used together [65].

The results of the present study failed to reveal a difference in the efficacy of PRF thermal stimulation compared to cupping therapy alone. The strongest explanation for our result was that the initial temperature setting was insufficient to achieve the heat-sensitive de-qi sensation on the APs. The PRF stimulation module used in this study was introduced by Myoung et al. [51, 66]. They compared the temperature distribution between moxibustion and subcutaneous PRF thermal stimulation with a maximum power of 200 W applied to an anaesthetized rabbit. The study showed that the two subcutaneous temperature distributions were highly correlated and that the rate of epidermal heat loss during PRF stimulation was less than the heat loss rate during moxibustion. Thus, PRF thermal stimulation may be more effective in maintaining heat intensities than moxibustion. However, the PRF module used in this study was designed not to generate heat above 42°C. In addition, the thermal stimulation applied in this trial was generated at only 10% of the maximum power (20 W) due to safety considerations because there is no existing evidence regarding the optimal safe intensity of PRF stimulation for human subjects for the treatment of LBP. Hence, use of the minimum intensity of PRF thermal stimulation was required in this trial, and the stimulation intensity chosen may be the main reason for the insignificant differences between the two different treatment groups. Similar results were found in other studies, and our study results are consistent with similar studies conducted by Lin et al. [67] and Shin et al. [53]. In those studies, there were no significant differences between the treatment group (laser acupuncture combined with cupping) and the placebo-controlled group (cupping), although significant differences in the VAS score within each treatment group were detected. The initial intensity of laser irradiation in both previous studies was a maximum power of 40-53 mW, wavelengths ranging from 660 to 808 nm, pulse frequencies of 20-200 Hz, a duty cycle of 50%, and 3 to 10 minutes of treatment. Regardless of the variation in laser dosage between the two studies, the length of infiltration was approximately 2-5 mm into the skin, which indicated that the intensity of stimulation was not sufficient to achieve the de-qi sensation: the optimal depth of acupuncture needle penetration has been suggested to be 10.3 to 90.3 mm [68]. Therefore, measurements of the de-qi sensation and further studies to identify the optimal intensity of electrically generated stimulation for humans are greatly needed for the development of convenient and safe medical devices to modulate CAM therapies for stimulating APs.

Another limitation of this study was that although the magnitude of negative pressure generated by the cupping instrument was not as strong as the pressure applied in conventional cupping therapy, its efficacy was not negligible. Recent studies reported that negative pressures generated from cupping therapy were ranged from 30 to 50 kPa [33, 69]. In contrast, low negative pressure (15 kPa) was applied in this study because the negative pressure was generated to adhere to the probes that were also used for PRF thermal stimulation in this stimulation system. Nonetheless, low-magnitude negative pressure is also widely used. In accordance with the survey of studies related to cupping therapy in South Korea performed in 2012, 50% of studies reported less than 100 mmHg (13.3 kPa) of cupping therapy, although the most frequently reported pressure was 600 mmHg (80 kPa) [70]. Moreover, the effectiveness of low negative pressure cannot be ignored because the results of previous and present studies have indicated that low-pressure cupping therapy is significantly effective in reducing LBP [53, 67]. However, with the exception of those two previous studies, no study has reported the effectiveness of low negative pressure stimulation. Therefore, further studies on the efficacy of low negative pressure applied during cupping therapy are required to confirm our results.

Finally, patients were informed that the PRF thermal stimulation would not be performed in the placebo-controlled group. Despite the fact that we originally employed the double-blinded design, patient blinding was probably broken due to the nature of the heat stimulus. Therefore, the study was suspected to be a single-blinded clinical trial. This limitation may lead to bias in the results of the study. In addition, the fact that the trial was registered retrospectively is another limitation of this study.

Moxibustion and cupping are popular CAM modalities used to manage LBP. Both modalities are typical adjuvants of acupuncture. Traditional moxibustion and cupping can cause adverse effects because heat and negative pressure are difficult to tolerate. Because sensitivity to stimulation varies among individuals, the development of a medical device enabling control of the intensity of stimulation is required. In this respect, the assessment of the efficacy of the device used in this present study is meaningful. To our knowledge, this is the first study to investigate the efficacy of PRF thermal stimulation for LBP through RCT and, therefore, the present study provides a guideline for future studies and for device development.

In conclusion, the present study shows the effectiveness of both PRF thermal stimulation plus cupping therapy and cupping therapy alone in reducing LBP, although there was no evidence indicating the efficacy of the PRF thermal stimulation applied here. Future studies will be focused on identifying the optimal PRF intensity for generating heat stimulation equivalent to that applied in moxibustion.

## Figures and Tables

**Table 1:** Baseline characteristics of the treatment and placebo-controlled groups.

	**Treatment (n=28)**	**Placebo-controlled (n=28)**	**p value**
***Baseline characteristics***			
**Sex**			
** Female**	21 (75.0)	23 (82.1)	0.775
** Male**	7 (25.0)	5 (17.9)	
**Age [years)**	47.86 (9.58)	43.93 (11.58)	0.172
**Duration of LBP [month)**	7.38 (44.57)	3.53 (11.20)	0.204†
**Exercise therapy during the study [count)**	3.00 (0.00)	3.00 (0.00)	0.668†
**BMI [kg/m** ^**2**^ **)**	23.81 (2.64)	24.77 (3.45)	0.245
**Systolic BP [mmHg]**	122.14 (14.18)	120.14 (14.16)	0.600
**Diastolic BP [mmHg)**	73.64 (11.04)	71.07 (9.03)	0.344
**Pulse [bpm]**	75.57 (10.64)	74.43 (10.81)	0.692
**Body temperature [**°**C)**	36.53 (0.53)	36.50 (0.41)	0.800
***Outcomes at baseline***			
**VAS [mm]**	41.96 (10.66)	46.25 (11.44)	0.153
**PPT (at visit 1) [kg/cm** ^**2**^ **]**	6.72 (1.87)	6.62 (1.68)	0.847
**EQ-5D (at visit 1)**	0.77 (0.09)	0.78 (0.08)	0.844

Data are summarized as the mean (standard deviation: SD) for the continuous variables and N (%) for the categorical variables. The p values were derived based on the independent two-sample t-test or Wilcoxon's rank sum test for the continuous variables and chi-squared test for the categorical variables.

†Derived from Wilcoxon's rank sum test.

Treatment: PRF-thermal stimulation plus cupping therapy; Placebo-controlled: cupping therapy.

BP: blood pressure; BMI: body mass index; VAS: visual analogue scale; PPT: pressure pain threshold; EQ-5D: Euro Quality of Life-5 Dimensions.

**Table 2:** Mean change compared to the baseline and the mean difference in change between the treatment and placebo-controlled groups at each visit for the primary and secondary outcomes.

	**Treatment**		**Placebo-controlled**		**Treatment–Placebo-controlled**	
	**Mean change from the baseline (95% CI)**	**p value**†	**Mean change from the baseline (95% CI)**	**p value**†	**Adjusted mean difference between the groups (95% CI)**	**p value**‡
***VAS *[mm]**						
Visit 1 – Baseline	0.357 (-3.448, 4.162)	0.9935	0.000 (-3.805, 3.805)	1.0000	0.118 (-0.384, 0.620)	0.6811
Visit 2 – Baseline	-2.857 (-6.662, 0.948)	0.3837	-3.036 (-6.841, 0.769)	0.3324	-1.299 (-5.949, 3.351)	0.2886
Visit 3 – Baseline	-5.000 (-8.805, -1.195)	0.0366	-9.107 (-12.912, -5.302)	<0.0001	1.826 (-4.664, 8.316)	0.7127
Visit 4 (F/U) – Baseline§	-8.036 (-11.841, -4.231)	0.0002	-13.393 (-17.198, -9.588)	<0.0001	2.015 (-5.288, 9.317)	0.7090
***PPT *[kg/cm** ^**2**^ **]**						
Visit 2 – Visit 1	-1.294 (-1.804, -0.784)	<0.0001	-0.878 (-1.388, -0.367)	0.0025	-0.581 (-1.308, 0.146)	0.0575
Visit 3 – Visit 1	-1.367 (-1.877, -0.857)	<0.0001	-1.020 (-1.530, -0.509)	0.0003	-0.417 (-1.087, 0.253)	0.1087
Visit 4 (F/U) – Visit 1§	-1.421 (-1.932, -0.911)	<0.0001	-0.935 (-1.445, -0.425)	0.0012	-0.507 (-1.217, 0.203)	0.0789
***PGIC***						
Visit 4 (F/U) – Visit 1§	-0.786 (-1.115, -0.457)	<0.0001	-0.929 (-1.258, -0.600)	<0.0001	0.053 (-0.419, 0.525)	0.5887
***EQ-5D***						
Visit 4 (F/U) – Visit 1§	0.026 (0.000, 0.052)	0.0485	0.050 (0.024, 0.076)	<0.0001	-0.022 (-0.056, 0.012)	0.9038

Data are summarized as the mean and 95% CI for the primary and secondary outcomes.

†Result of the within-groups analysis using a linear mixed effects model; p values were adjusted with Dunnett's test.

‡Result of the between-groups analysis using an ANCOVA with the following covariates: the baseline value, age, patient's duration of LBP, and compliance of daily exercise.

§Follow-up end point.

CI: confidence interval; VAS: visual analogue scale; PPT: pressure pain threshold; PGIC: patient global impression of change; EQ-5D: Euro Quality of Life-5 Dimensions.

## Data Availability

The clinical data used to support the findings of this study are available from the corresponding author upon request.

## List of Abbreviations

PRF: Pulsed radiofrequency
AP: Acupoints
LBP: Low back pain
CAM: Complementary and alternative medicine
TCM: Traditional Chinese medicine
RCT: Randomized control trial
VAS: Visual analogue scale
PPT: Pressure pain threshold
PGIC: The patient global impression of change
EQ-5D: European quality of life-5 dimensions
FAS: Full analysis set
ANCOVA: Analysis of covariance.
